# Structural and functional changes of anterior cingulate cortex subregions in migraine without aura: relationships with pain sensation and pain emotion

**DOI:** 10.1093/cercor/bhae040

**Published:** 2024-02-10

**Authors:** Yangxu Ou, Xixiu Ni, Xiaoyu Gao, Yang Yu, Yutong Zhang, Yanan Wang, Jie Liu, Zihan Yin, Jing Rong, Mingsheng Sun, Jiao Chen, Zili Tang, Wang Xiao, Ling Zhao

**Affiliations:** Acupuncture and Tuina School, Chengdu University of Traditional Chinese Medicine, No. 1166, Liutai Avenue, Wenjiang District, Chengdu, Sichuan 611137, China; Acupuncture and Tuina School, Chengdu University of Traditional Chinese Medicine, No. 1166, Liutai Avenue, Wenjiang District, Chengdu, Sichuan 611137, China; Acupuncture and Tuina School, Chengdu University of Traditional Chinese Medicine, No. 1166, Liutai Avenue, Wenjiang District, Chengdu, Sichuan 611137, China; Acupuncture and Tuina School, Chengdu University of Traditional Chinese Medicine, No. 1166, Liutai Avenue, Wenjiang District, Chengdu, Sichuan 611137, China; Department of Scientific Research and Education and Training Management, the Third People’s Hospital of Chengdu, Chengdu, Sichuan 610000, China; Department of Pain Treatment, Chongqing Hospital of Traditional Chinese Medicine, Chongqing 400021, China; Department of Neurology, Sichuan Provincial People’s Hospital, Chengdu, Sichuan 610072, China; Acupuncture and Tuina School, Chengdu University of Traditional Chinese Medicine, No. 1166, Liutai Avenue, Wenjiang District, Chengdu, Sichuan 611137, China; Acupuncture and Tuina School, Chengdu University of Traditional Chinese Medicine, No. 1166, Liutai Avenue, Wenjiang District, Chengdu, Sichuan 611137, China; Acupuncture and Tuina School, Chengdu University of Traditional Chinese Medicine, No. 1166, Liutai Avenue, Wenjiang District, Chengdu, Sichuan 611137, China; Sichuan Clinical Medical Research Center for Acupuncture and Moxibustion, Chengdu, Sichuan 611137, China; Acupuncture and Tuina School, Chengdu University of Traditional Chinese Medicine, No. 1166, Liutai Avenue, Wenjiang District, Chengdu, Sichuan 611137, China; Sichuan Clinical Medical Research Center for Acupuncture and Moxibustion, Chengdu, Sichuan 611137, China; Acupuncture and Tuina School, Chengdu University of Traditional Chinese Medicine, No. 1166, Liutai Avenue, Wenjiang District, Chengdu, Sichuan 611137, China; Acupuncture and Tuina School, Chengdu University of Traditional Chinese Medicine, No. 1166, Liutai Avenue, Wenjiang District, Chengdu, Sichuan 611137, China; Acupuncture and Tuina School, Chengdu University of Traditional Chinese Medicine, No. 1166, Liutai Avenue, Wenjiang District, Chengdu, Sichuan 611137, China; Sichuan Clinical Medical Research Center for Acupuncture and Moxibustion, Chengdu, Sichuan 611137, China

**Keywords:** migraine without aura, anterior cingulate cortex, functional connectivity, gray matter volume

## Abstract

Migraine without aura is a multidimensional neurological disorder characterized by sensory, emotional, and cognitive symptoms linked to structural and functional abnormalities in the anterior cingulate cortex. Anterior cingulate cortex subregions play differential roles in the clinical symptoms of migraine without aura; however, the specific patterns and mechanisms remain unclear. In this study, voxel-based morphometry and seed-based functional connectivity were used to investigate structural and functional alterations in the anterior cingulate cortex subdivisions in 50 patients with migraine without aura and 50 matched healthy controls. Compared with healthy controls, patients exhibited (1) decreased gray matter volume in the subgenual anterior cingulate cortex, (2) increased functional connectivity between the bilateral subgenual anterior cingulate cortex and right middle frontal gyrus, and between the posterior part of anterior cingulate cortex and right middle frontal gyrus, orbital part, and (3) decreased functional connectivity between the anterior cingulate cortex and left anterior cingulate and paracingulate gyri. Notably, left subgenual anterior cingulate cortex was correlated with the duration of each attack, whereas the right subgenual anterior cingulate cortex was associated with migraine-specific quality-of-life questionnaire (emotion) and self-rating anxiety scale scores. Our findings provide new evidence supporting the hypothesis of abnormal anterior cingulate cortex subcircuitry, revealing structural and functional abnormalities in its subregions and emphasizing the potential involvement of the left subgenual anterior cingulate cortex-related pain sensation subcircuit and right subgenual anterior cingulate cortex -related pain emotion subcircuit in migraine.

## Introduction

Migraine without aura (MWoA) is a common and highly prevalent neurological disorder characterized by unilateral, throbbing, and pulsating headaches associated with photophobia, phonophobia, nausea, and vomiting (May and Schulte 2016; Minen et al. 2016; Ashina et al. 2021; Khan et al. 2021; Gerstein et al. 2023). More and more studies have shown that migraine is more than just a headache, involving altered processing of sensory and negative stimuli (Szabó et al. 2019; Kim et al. 2021; Gerstein et al. 2023). Prolonged and repeated attacks of pain can seriously affect the daily activities of patients and exacerbate various symptoms such as vomiting, nausea, and photophobia (Szabó et al. 2019; Li et al. 2023). Pain-associated affective (such as depression and anxiety) disorders can further contribute to headache-related disabilities (Wolf et al. 2020). Frequent attacks of MWoA can exacerbate pain and elevate the heightened risk of physical diseases (such as stroke and unstable angina; (Vgontzas and Pavlović 2018; Øie et al. 2020) and psychological comorbidity (such as anxiety and fear) (Charles and Baca 2013), thereby deteriorating the patients’ health and quality of life. Additional research has shown that pain sensation and emotions in patients with MWoA are linked to abnormal activity of the anterior cingulate cortex (ACC; Bussone et al. 2012; Palomero-Gallagher et al. 2019; Wang et al. 2022a). Amounts of studies have focused on the specific associations between migraine pain sensation, pain emotions, and the ACC. Nevertheless, the specific neuroimaging mechanisms underlying these effects remain unknown.

The ACC plays a vital role in integrating and regulating the multidimensional clinical manifestations of migraines (Lee et al. 2022; Mao et al. 2022; Journée et al. 2023). Neuroimaging techniques have been widely utilized to investigate changes in structure and function in the ACC during migraines (Liu et al. 2015; Juarez-Salinas et al. 2019; Wang et al. 2022b; Yang et al. 2022). For example, voxel-based morphometric (VBM) analysis has found a decreased gray matter volume (GMV) in the left dorsal ACC (dACC) or right ACC, which is correlated with the migraine duration and Visual Analog Scale (VAS) scores, respectively (Jin et al. 2013; Wang et al. 2022c). Local functional activity studies have shown that decreased amplitude low-frequency fluctuation (ALFF) values of the rostral ACC (rACC) may be correlated with the frequency of migraine attacks, and decreased regional homogeneity (ReHo) values are negatively associated with the duration of disease in the ACC (Xue et al. 2013; Zhao et al. 2013). Moreover, researches on functional connectivity (FC) and brain networks have demonstrated decreased FC between the left ACC and thalamus, which was negatively associated with headache impact test score in MWoA patients. Additionally, the increased FC between the subgenual ACC (sACC)/perigenual ACC (pACC) and the angular gyrus was linked to the self-rating anxiety scale (SDS) scores. Furthermore, the decreased FC between the pACC and ventromedial prefrontal cortex was linked to migraine severity (Yuan et al. 2012, 2013; Androulakis et al. 2018; Argaman et al. 2020; Chen et al. 2022; Mao et al. 2022; Yang et al. 2022). The inconsistencies of the above findings may be attributed to variations in the distinct contributions of ACC subregions and a lack of systematic structural and functional studies. Previous researches have demonstrated that the ACC is heterogeneous in structure and function, and ACC subregions have different roles, with the sACC involved in emotion regulation and cognitive regulation (Ashburner and Friston 2000; Margulies et al. 2007; Kelly et al. 2009). Therefore, there is an urgent need to systematically integrate the association between structural and functional changes of the ACC subregions and the clinical manifestations of migraine.

In the current study, we combined structural and functional analyses to explore the structural and functional alterations in the ACC in MWoA and further assess the contributions of these changes to various clinical symptoms in patients with MWoA. We hypothesized that structural and functional alterations in the ACC vary in different subdivisions or subcircuits, and that these abnormalities might be linked to pain sensation and pain emotions in migraineurs.

## Material and methods

### Study population and protocol

We recruited patients who were diagnosed with MWoA between May 2020 and December 2022 at the Hospital of Chengdu University of Traditional Chinese Medicine (TCM) and Sichuan Provincial People’s Hospital. This study adhered to the principles of the Declaration of Helsinki, received approval from the Ethics Committee of the Hospital of Chengdu University of TCM (Ethics Approval No. 2020KL-003), and was registered with the China Clinical Trial Registry (Registration No. ChiCTR2000032308). The study was presented to or explained to each participant, either verbally or in writing. The participants were asked about their thoughts and provided informed consent by signing an informed consent form.

### Participants

Fifty eligible were recruited from the Chengdu University of TCM and Sichuan Provincial People’s Hospital. The diagnostic criteria for MWoA were adopted from the Headache Classification Committee of the Headache Society, Third Edition (2018). Individuals who fulfilled the following five inclusion criteria were included: (1) right-handed; (2) two to eight migraine attacks per month in the past three months and during the baseline period (4 weeks before enrollment); (3) 18–55 years of age; (4) onset of headache before 50 years of age; and (5) informed consent signed by the participant himself/herself or on behalf of the patient by his/her immediate family members. The exclusion criteria were (1) those with neurologic diseases; (2) those with cardiovascular disease, diabetes mellitus, hypertension, hyperlipidemia, or any primary systemic disease; (3) pregnant and lactating women, and those who had a request for conceiving in the last six months; (4) those with a history of alcoholism or drug abuse; (5) being enrolled in other clinical trials; (6) the patient had other contraindications in magnetic resonance imaging (MRI) examination, such as claustrophobia; and (7) the patient was unable to understand the instructions given by the physicians.

In addition, 50 healthy individuals who were right-handed and fulfilled the age and education criteria were recruited from the community as healthy controls (HCs). The participants and their family had not experienced migraines or headaches.

### Clinical assessments

In this study, participants’ age, gender, weight, height, education, pain medication use, duration of illness, duration of each attack, self-rating anxiety scale (SAS), VAS, SDS, and migraine-specific quality-of-life questionnaire (MSQ [emotion, restriction, preventability]) scores were recorded. Pain sensation was clinically evaluated using a VAS, and the duration of each attack was noted in hours (2018; Özçelik et al. 2022). Pain emotion was clinically assessed using the MSQ (emotion), SAS, and SDS (Sengul et al. 2018; Ailani et al. 2021; Duan et al. 2023). The MSQ (restriction) and MSQ (preventability) are mainly used to assess pain-induced functional limitations.

### fMRI data acquisition

A 3.0 T MRI scanner (GE, Discovery MR750) was used to acquire the functional MRI (fMRI) images at the MRI Center, Hospital of Chengdu University of TCM. During MRI scanning, all participants were expected to remain as still as possible with their eyes closed while keeping their minds free of thoughts. Subjects received verbal instructions before scanning and were asked afterwards if they had fallen asleep during the scanning process. Functional images were acquired laterally using echo-planar imaging with the following parameters: TR/TE = 2000 ms/30 ms, flip angle = 90°, 40 slices, 64 × 64 matrices, field of view = 250 × 250 mm^2^, slice gap = 0 mm, voxel size = 3.75 × 3.75 × 5 mm^3^. The scan lasted for 6 min for each subject, and 180 functional volumes were obtained. If a patient experienced a migraine attack during the scanning process, the scanning was stopped immediately, and the scanning time was postponed.

### fMRI data processing

We used DPABI V7.0 (http://rfmri.org/DPABI) to preprocess brain imaging data (Yan et al. 2016; Wang et al. 2022a). The procedure involved discarding the first five volumes, followed by calibration of the remaining images. The maximum head motion on each axis was checked for movements < 2 mm or rotations > 2.0°, to ensure that there were no unexpected head movements. Data were converted into EPI templates (resampled voxel size, 3 × 3 × 3 mm^3^). Covariates (including white matter signal, friction 24 motor parameters, and cerebrospinal fluid) were regressed. No global signal regression was performed (Wang et al. 2019). Finally, detrending and bandpass filtering were performed.

### GMV analysis

Structural T1-weighted images were preprocessed and statistically analyzed using the VBM8 toolbox in SPM8 (http://dbm.neuro.uni-jena.de/vbm8). Furthermore, the acquired images of each subject were uniformly non-linearly transformed and resampled using the Montreal Neurological Institute (MNI) template and resampled to a voxel size of 1.5 × 1.5 × 1.5 mm^3^. The normalized images were segmented into gray matter, white matter, and cerebrospinal fluid. The data were further kernelled and smoothed using a 6 mm full width at half-maximum Gaussian kernel (Ashburner and Friston 2000).

### FC analyses

#### Seed generation

The specific steps of seed point segmentation or generation were reported in reference to previous literature with the following considerations (Margulies et al. 2007; Kelly et al. 2009). First, based on anatomical and neuroimaging findings the ACC subregion can be distinguished into functionally and structurally distinct subregions (Lütcke and Frahm 2008; Kelly et al. 2009; Peng et al. 2021). Second, the generation of seed points mainly included the following process. Firstly, two parallel spherical seed arrays (123 voxels in 1 × 1 × 1 mm^3^ space, radius = 3 mm) were created, and then lined up in the ACC according to the following steps: (1) tracing the corpus callosum and creating two parallel curves in the ACC: the corpus callosum was traced on a standard 152-brain MNI template, and a quadratic function was used to fit it; using the corpus callosum curve as a reference, two parallel curves were created within the ACC: one is 5 mm above the corpus callosum curve (“lower”), and the other is 15 mm above the corpus callosum curve (“upper”). (2) Seed point coordinate generation: nine equidistant (10 mm distance) points were calculated starting from *y* = −10 mm; seven points on the upper curve were calculated, and each point was distributed radially from the lower point, creating a set of seeds for each hemisphere at *x* = ± 5 mm. The five points with the most uniform distribution and the greatest structural and functional differentiation were finally selected as seed points (Peng et al. 2021), which formed the basis for the subdividing of the ACC into subregions in our study.

#### Seed selection and analysis

Based on previous researches on ACC subdivision (Margulies et al. 2007; Kelly et al. 2009), the coordinates of seed points in each hemispheric ACC subregion were selected as follows: sACC (L/R, light purple, and MNI = ± 5, 34, −4), pACC (L/R, dark purple, and MNI = ± 5, 47, 11), rACC (L/R, blue, and MNI = ± 5, 27, 21), caudal ACC (cACC [L/R, red, and MNI = ± 5, −10, 37]), dACC (L/R, orange, and MNI = ± 5, 10, 33). The aforementioned seed points of the ACC were distributed within a radius of 3 mm in the bilateral hemispheres, and based on previous anatomical and functional images of the ACC, more pronounced heterogeneity of ACC structure and function has been detected (Paus 2001; Margulies et al. 2007; Peng et al. 2021; Zhang et al. 2022). The combined volume–surface analysis method was employed to calculate FC maps for each region of interest within the ACC subregions. The MNI volume space was used to average each seed point within the ACC subregion across all voxels. The FC of all the vertices of the participants was calculated in the cortical surface space by averaging the signals of each seed point. Finally, correlation plots of the Fisher’s *r*-to-*z* transformations were averaged over the group of seed points in each ACC subregion to create average FC plots.

### Statistical analysis

Clinical baseline data were statistically analyzed using SPSS (version 25.0; SPSS Inc., Chicago, IL, USA), and imaging data were analyzed using MATLAB R2016b. The baseline characteristics of the MWoA and HC groups were determined using descriptive statistics. Two-tailed χ^2^ tests and two-sample *t*-tests were performed for comparisons between groups.

Voxel-wise between-group analyses were performed for ACC subregions using two-sample *t*-test to identify abnormal FC differences between the MWoA and HC groups. The correction method was Gaussian Random Field correction (voxels, *P* < 0.01; cluster, *P* < 0.05, cluster size > 50 voxels). Furthermore, the association between the clinical symptoms of MWoA (VAS, duration of each attack, MSQ [emotion], SAS, and SDS) and FC was investigated using correlation analysis. *P*-values < 0.05 were considered statistically significant.

## Results

After screening 108 participants, five participants were excluded due to failure to meet the inclusion or exclusion criteria, and three participants were excluded due to head motion and missing data acquisition. Overall, 100 participants, including 50 patients with MWoA and 50 HCs, were included in the final analysis (Supplementary Fig. 1).

### Patient characteristics

There were no significant differences were found between the MWoA and HC groups regarding baseline characteristics (age, weight, height, education, gender, pain medication use, and duration of illness) (*P* > 0.05) (Table 1).

**Table 1:** Participant demographic and baseline characteristics.

	MWoA (*N* = 50)	HC (*N* = 50)	*P*-value
Age, mean (SD), y	36.82 (11.23)	34.62(6.21)	0.31^a^
Gender (male/female)	16/34	23/27	0.29^b^
Weight, mean (SD), kg	57.16 (10.51)	56.82 (7.91)	0.69^a^
Height (SD), cm	159.43 ± 4.32	159.31 ± 5.52	0.89^a^
Education, mean (SD), y	12.93 (4.02)	12.23 (4.23)	0.87^b^
Duration of illness, mean (SD), y	10.00(7.91)	—	—
Pain medication use (y/n)	2/48	-	-
VAS scores, mean (SD)	5.76 (1.90)	—	—
Duration of each attack, mean (SD), h	13.93 (12.51)	—	—
MSQ (resistance), mean (SD)	61.48 (20.27)	—	—
MSQ (preventability), mean (SD)	74.32 (20.52)	—	—
MSQ (emotion), mean (SD)	74.79 (21.05)	—	—
SAS, mean (SD)	44.94 (8.90)	—	—
SDS, mean (SD)	42.58 (10.39)	—	—

Abbreviations: MWoA, migraine without aura; HC, healthy control; SD, standard deviation; VAS, visual analog scale; MSQ (resistance), migraine-specific quality-of-life questionnaire (role restrictive); MSQ (preventability), migraine-specific quality-of-life questionnaire (role preventive); MSQ (emotion), migraine-specific quality-of-life questionnaire (emotional function); SAS, self-rating anxiety scale; SDS, self-rating depression scale.

*P* ^a^-value was obtained by χ^2^ two-tailed test.

*P* ^b^-value was obtained by two-sample *t*-test.

### Voxel-based morphometry results

**Fig. 1:**
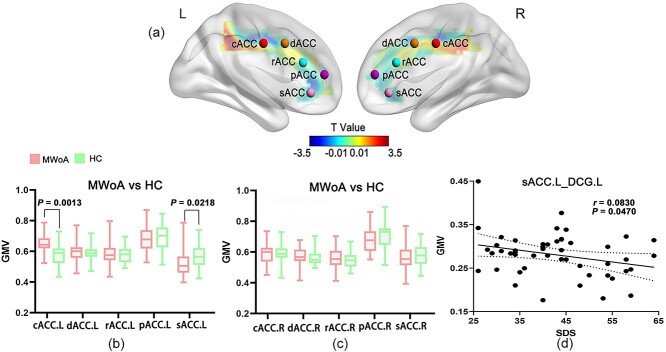
Structural analysis results of patients with MWoA compared with HCs. (a) GMV distribution in bilateral ACC subregions in MWoA patients, with warm colors indicating increased GMV in the subregions and cool colors indicating decreased GMV in the subregions; changes in the GMV in left (b) and right (c) ACC subregions in MWoA patients compared with HCs. *P* < 0.05 indicates statistically significant differences; (d) decreased GMV between the sACC.L and DCG.L was negatively correlated with SDS scores (*P* = 0.0470). Abbreviations: MWoA, migraine without aura; HC, healthy control; GMV, gray matter volume; cACC, caudal anterior cingulate cortex; dACC, dorsal anterior cingulate cortex; rACC, rostral anterior cingulate cortex; pACC, perigenual anterior cingulate cortex; sACC, subgenual anterior cingulate cortex; SDS, self-rating depression scale; DCG.L, left median cingulate and paracingulate gyri; R, right; L, left.

Changes in the VBM of the ACC in patients with MWoA can be divided into two regions: anterior and posterior (Fig. 1). Specifically, patients with MWoA exhibited decreased GMV in the anterior part of ACC (e.g. sACC.L) (*P* = 0.0218) and increased GMV in the posterior part of ACC (e.g. cACC.L) (*P* = 0.0013), as compared to HCs.

Furthermore, the decreased GMV between the left median cingulate and paracingulate gyri (DCG.L) and the sACC.L in patients with MWoA was negatively linked to SDS scores (*r* = 0.0830, *P* = 0.0470).

### FC results

#### FC maps of each ACC subregion

**Fig. 2:**
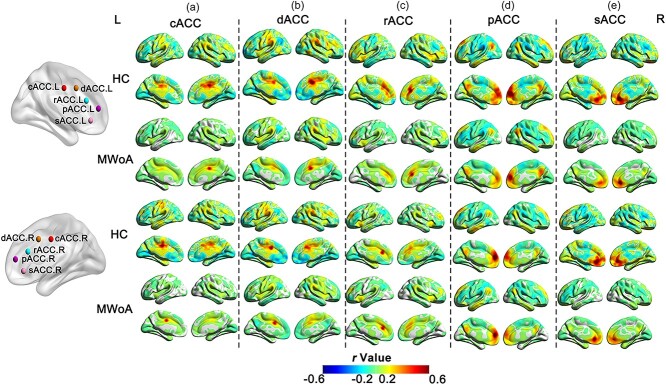
FC maps of 10 ACC subregions in MWoA patients (*n* = 50) and HCs (*n* = 50). (a) FC map of MWoA patients and HCs with bilateral cACC([L/R, red, and MNI = ± 5, −10, 37]) as seed points; (b) FC map of MWoA patients and HC with bilateral dACC(L/R, orange, and MNI = ± 5, 10, 33) as seed points; (c) FC map of MWoA patients and HC with bilateral rACCs (L/R, blue, and MNI = ± 5, 27, 21) as seed points; (d) FC map of MWoA patients and HC with bilateral pACCs (L/R, dark purple, and MNI = ± 5, 47, 11) as seed points; (e) FC map of MWoA patients and HC with bilateral sACCs (L/R, light purple, and MNI = ± 5, 34, −4) as seed points. All seeds were identified based on MNI coordinates within a radius of 3 mm. Abbreviations: FC, functional connectivity; MWoA, migraine without aura; HC, healthy control; MNI, Montreal neurological institute; cACC, caudal anterior cingulate cortex; dACC, dorsal anterior cingulate cortex; rACC, rostral anterior cingulate cortex; pACC, perigenual anterior cingulate cortex; sACC, subgenual anterior cingulate cortex; R, right; L, left.

The ACC subregion seeds in the bilateral hemispheres exhibited similar FC patterns in the MWoA and HC groups (Fig. 2). Taking the left hemisphere of the ACC as an example, the cACC is mainly connected to the sensorimotor network (SMN) and superior parietal lobe (SPL). The dACC is predominantly linked to the dorsolateral prefrontal cortex (dlPFC), insula, and supplementary motor cortex, whereas the rACC is connected to the prefrontal lobes, insula, and dlPFC. Furthermore, the pACC was mainly linked to the IPL and posterior cingulate cortex (PCC), whereas the sACC was mainly linked to the limbic areas and orbitofrontal cortex regions.

### Between-group differences in the FC of the ACC subregions of MWoA patients and HCs

**Fig. 3:**
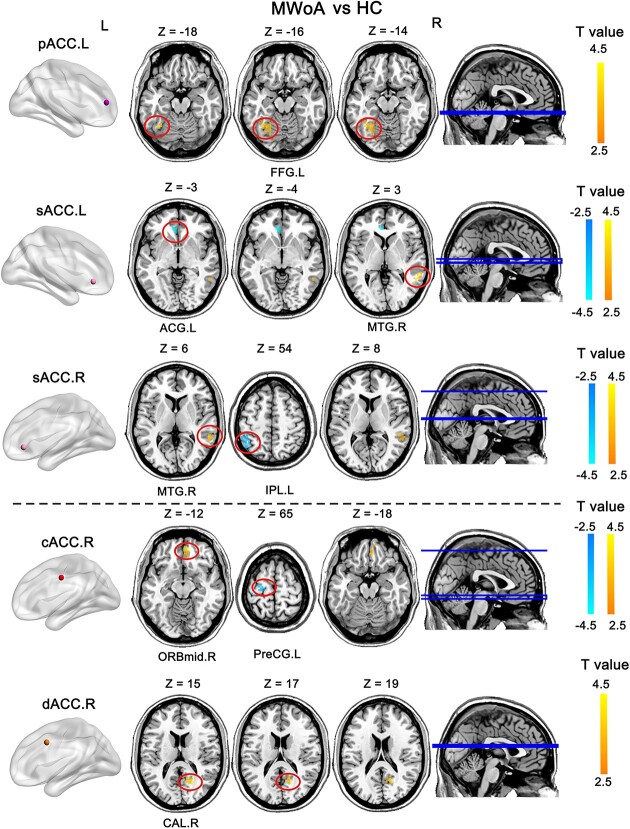
Altered FC of ACC subregions in patients with MWoA compared with HCs. Warm colors indicate increased FC between sub-regions and brain regions in patients with MWoA, while cool colors indicate decreased FC in patients with MWoA. Abbreviations: FC, functional connectivity; MWoA, migraine without aura; HC, healthy control; cACC, caudal anterior cingulate cortex; dACC, dorsal anterior cingulate cortex; rACC, rostral anterior cingulate cortex; pACC, perigenual anterior cingulate cortex; sACC, subgenual anterior cingulate cortex; R, right; L, left. FC, functional connectivity; MTG.R, right middle temporal gyrus; ACG.L, left anterior cingulate and paracingulate gyri; FFG.L, left fusiform gyrus; IPL.L, left inferior parietal; PreCG.L, left precentral gyrus; ORBmid.R, right middle frontal gyrus, orbital part; CAL.R, right calcarine.

Patients with MWoA exhibited increased FC between the bilateral sACC (sACC.R and sACC.L) and the right middle frontal gyrus (MTG.R) and decreased FC within the left anterior cingulate and paracingulate gyri (ACG.L), compared with HCs (Fig. 3 and Table 2). Furthermore, patients with MWoA exhibited increased FC between the posterior part of ACC (dACC.R and cACC.R) and the right middle frontal gyrus and orbital part (ORBmid.R), and decreased FC with the left precentral gyrus (PreCG.L). Specifically, patients with MWoA showed increased FC between the cACC.R and ORBmid.R, decreased FC between cACC.R and the PreCG.L, and increased FC between the dACC.R and right calcarine (CAL.R), as compared with HCs. Furthermore, patients with MWoA showed increased FC between the sACC.L and MTG.R, and decreased FC in the ACG.L. Additionally, patients with MWoA exhibited increased FC in the pACC.L and left fusiform gyrus (FFG.L) compared with HCs.

**Table 2:** Brain regions showing a significant difference between MWoA and HC.

	ROIs	Brain areas	Cluster size voxels	Peak value	Peak coordinate (MNI)		
					X	Y	Z
MWoA < HC	sACC.L	ACG.L	90	−8.10	−6	33	0
	cACC.R	PreCG.L	54	−3.60	54	−27	−51
	sACC.R	IPL.L	87	−3.33	−45	−48	54
MWoA > HC	pACC.L	FFG.L	121	4.01	−36	−57	−18
	sACC.L	MTG.R	102	4.09	57	−15	3
	dACC.R	CAL.R	63	4.48	18	−60	15
	sACC.R	MTG.R	73	3.94	−60	−36	3
	cACC.R	ORBmid.R	72	3.79	9	51	−12

Abbreviations: ROI, region of interest; MNI, Montreal Neurological Institute; MWoA, migraine without aura; HC, healthy control; cACC, caudal anterior cingulate cortex; dACC, dorsal anterior cingulate cortex; rACC, rostral anterior cingulate cortex; pACC, perigenual anterior cingulate cortex; sACC, subgenual anterior cingulate cortex; R, right; L, left. ACG.L, left anterior cingulate and paracingulate gyri; PreCG.L, left precentral gyrus; IPL.L, left inferior parietal; FFG.L, left fusiform gyrus; MTG.R, right middle temporal gyrus; CAL.R, right calcarine; ORBmid.R, right middle frontal gyrus, orbital part.

**Fig. 4:**
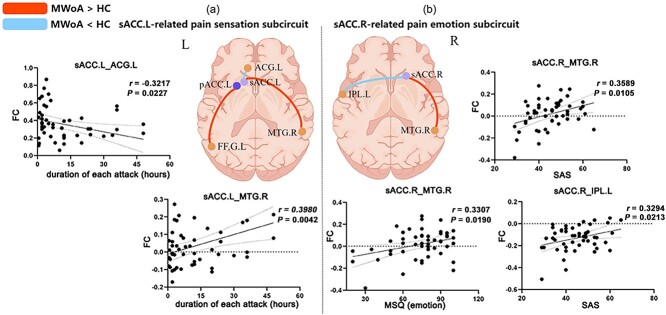
Correlation analysis of ACC subregion-related circuits and clinical symptoms in MWoA. (a) Schematic brain map illustrating the sACC.L-related pain sensation sub-circuit; correlation analysis between the sACC.L-ACG.L and MTG.R and the duration of each attack. (b) Schematic brain map showing the sACC.L-related pain emotion subcircuit; correlation analysis between the sACC.R-MTG.R and IPL.L and SAS and MSQ (emotion) scores. Abbreviations: MWoA, migraine without aura; HC, healthy control; sACC, subgenual anterior cingulate cortex; pACC, perigenual anterior cingulate cortex; R, right; L, left. MTG.R, right middle temporal gyrus; ACG.L, left anterior cingulate and paracingulate gyri; IPL.L, left inferior parietal; FFG.L, left fusiform gyrus; MSQ (emotion), migraine-specific quality-of-life questionnaire (emotional function); SAS, self-rating anxiety scale.

Surprisingly, we also found different patterns of FC in the bilateral anterior part of ACC. Specifically, patients exhibited increased FC between the sACC.L and MTG.R, and decreased FC within the ACG.L. Patients exhibited increased FC between the sACC.R and MTG.R and decreased FC within the IPL.L (Fig. 4).

### Correlation between FC and clinical symptoms in MWoA

Patients with MWoA exhibited significantly increased FC between the sACC.L and MTG.R, which was positively correlated with the duration of each attack (*r* = 0.3980, *P* = 0.0042). However, patients with MWoA also exhibited increased FC between the sACC.L and ACG.L, which was inversely linked to the duration of each attack (*r* = −0.3217, *P* = 0.0227). Furthermore, FC between the sACC.R, MTG.R, and IPL.L was positively linked to MSQ (emotion) (*r* = 0.3307, *P* = 0.0190), SAS (*r* = 0.3589, *P* = 0.0105), and SAS (r = 0.3294, *P* = 0.0213) scores (Fig. 4).

## Discussion

To the best of our knowledge, this study is the first to unveil the neuroimaging foundation of pain sensation and emotion in patients with MWoA by examining structural and functional alterations in the ACC subregions. Our findings confirmed the heterogeneity of structural and functional alterations in the ACC subregions among patients with MWoA. First, patients showed that anterior and posterior GMV changes in the ACC subregions are inconsistent, specifically, decreased GMV in the sACC and increased GMV in the cACC in MWoA patients. Second, MWoA patients exhibited different patterns of FC in the anterior and posterior parts; specifically, increased FC between the bilateral sACC and the middle default mode network (DMN [MTG.R]), between the cACC.R and anterior DMN (ORBmid.R), as well as the decreased FC between the sACC.L and salience network (SN [ACG.L]), compared with HCs. Interestingly, there are differences between the left and right anterior part of ACC; in particular, FC of the sACC.L is linked to the duration of each attack, while that of the sACC.R is associated with MSQ (emotion) and SAS scores. Our findings provide specific views on variations in pain sensation and pain emotion in patients with MWoA, emphasizing the fundamental contributions of the sACC.L-related pain sensation and sACC.R-related pain emotion subcircuits to migraine.

### Alterations in the GMV of ACC subdivisions in MWoA

In this study, we observed a decrease in GMV in the anterior part of ACC (e.g. sACC) and increased GMV in the posterior part of ACC (e.g. cACC) of MWoA patients compared with HCs. Previous researches have demonstrated that changes in the GMV of the ACC are closely associated with pain management in patients with migraine (Jin et al. 2013; Russo et al. 2020; Yu et al. 2021). Our previous study suggested that decreased GMV of the sACC/pACC in patients with menstrual migraine (MM) correlated with VAS and SDS scores (Wang et al. 2022b). The ACC forms a major part of the limbic system and is involved in various sensory and emotional processes. One study has shown that the ACC is heterogeneous in structure and function (Palomero-Gallagher et al. 2019). Migraine has a different prevalence in men and women, and persistent chronic pain inevitably leads to psychological and psychiatric changes with negative emotions, which are more pronounced in female patients(Wiech and Tracey 2009; van Casteren et al. 2021). Additionally, studies had found that female migraineurs exhibited reduced GMV in the ACC and prefrontal cortex, which is consistent with the findings of the present study(Dai et al. 2015; Wang et al. 2022a). The anterior part of ACC mainly includes the sACC and pACC, whose primary functions are emotional regulation and social processing. Our previous research demonstrated that GMV changes in the pACC were linked to the frequency of pain crises and the occurrence of depression (Wang et al. 2022b). The posterior part of ACC mainly comprises the cACC and dACC, whose main functions are sensorimotor processing and cognitive control, respectively. Researches have exhibited that a decrease in GMV of the dACC is related to pain duration and cognitive function in MWoA (Jin et al. 2013; Gao et al. 2016; Russo et al. 2020). Consequently, the heterogeneity in the distribution of ACC subregions among patients with MWoA corresponds to functional differences. This insight will aid in understanding the distinct contributions of ACC subregions to various clinical symptoms in MWoA.

### Abnormal FC from the anterior part of ACC to the middle DMN and SN, and from the posterior part of ACC to the anterior DMN and SMN

Our current findings showed increased FC between the anterior part of ACC (sACC and pACC) and middle DMN (MTG), and decreased FC between the sACC and SN (ACG). Our previous study showed abnormalities in the ACC-DMN pattern network in patients with MM (Wang et al. 2022a). Additionally, researches have demonstrated that patients with MWoA exhibit abnormal connections between the ACC and multiple networks (including the DMN, SN, and SMN), and these abnormalities are linked to subjective pain and negative emotions (Farmer et al. 2012; Veréb et al. 2020; Wang et al. 2022b). The DMN is primarily composed of the lateral parietal lobe, prefrontal cortex, and medial temporal lobe, and is associated with cognitive activity, emotion, and pain sensation (Andrews-Hanna et al. 2010; Raichle. 2015). A previous study found that the DMN was strongly correlated with pain sensation, anxiety, and depression in patients with migraine (Farmer et al. 2012). The MTG is a multisensory associative region and a crucial part of the DMN that plays an essential role in distributing the emotional tones of short-term memory associated with pain (Olson et al. 2007; Asari et al. 2008). Our previous study suggested that changes in ALFF and ReHo values in the MTG are related to pain intensity and disease duration in MWoA, consistent with our current findings (Zhao et al. 2013; Zhang et al. 2021). Furthermore, the SN is mainly composed of the ACC, the anterior insula, whereas the ACG is part of the ACC. The SN plays an essential role in the regulation of cognition, emotions, and pain (Schimmelpfennig et al. 2023). It has been demonstrated that abnormal FC within the SN, which is composed of the ACC-ACG circuitry, is linked to the frequency of migraine attacks (Veréb et al. 2020; Dai et al. 2021). Therefore, these findings indicate an imbalanced state in MWoA patients characterized by increased FC between the anterior part of ACC and middle DMN or decreased FC between the anterior part of ACC and SN, which mainly reflects pain sensation, mood, and cognition-related disorders.

In addition, our study demonstrated that patients with MWoA exhibited increased FC between the posterior part of ACC (dACC.R and cACC.R) and the anterior DMN (ORBmid.R) and decreased FC within the SMN (PreCG.L). ORBmid is located within the prefrontal cortex and is part of the DMN. Previous researches have exhibited that aberrant connections between the medial prefrontal cortex and the ACC, PCC, and insula are closely associated with migraine severity, pain cognition, and emotion regulation (Kong et al. 2013; Mathur et al. 2015). Decreased FC between the dACC and prefrontal cortex in patients with migraine is associated with pain severity (Androulakis et al. 2018). Furthermore, the dACC, as part of the posterior part of ACC, plays a crucial role in cognitive control. The cingulo-opercular network, consisting of the dACC and bilateral inferior frontal gyrus, supports cognitive persistence (Teubner-Rhodes. 2020). In addition, the PreCG was significantly associated with sensorimotor function. Several studies have shown that pain-related diseases are often accompanied by sensorimotor abnormalities (Zhang et al. 2017; Wei et al. 2020). During intense pain, the simultaneous activity of the cACC and sensorimotor-related networks supports the idea that brain networks are simultaneously active in pain sensation and cognition (Seminowicz and Davis 2007). Moreover, a previous study has shown increased FC between the dACC and sensorimotor areas in older adults, suggesting that the ACC and SMN are synergistically involved in processes such as sensorimotor integration and attentional control (Lin et al. 2017). Consequently, the above findings indicated that the increase FC between the posterior part of ACC and anterior DMN, or the decrease FC between the posterior part of ACC and SMN, was in an imbalanced state, primarily reflecting pain emotion, and sensorimotor-related disorders.

### The altered sACC.L-related pain sensation subcircuit and sACC.R-related pain emotion subcircuit

We performed a correlation analysis to further elucidate the pathophysiological mechanisms underlying the alterations in FC within the ACC subregions in MWoA. Correlation analysis results indicated that the left and right sACC differentially contribute to the integration and modulation of migraine pain sensations and pain emotions. Our study found that the left sACC was associated with pain sensation (the duration of each attack). Furthermore, researches have suggested that the left sACC is a key region for pain activation and may be closely related to pain sensation in migraine, which is consistent with our findings (Schulz-Stübner et al. 2004; Mao et al. 2022). It is well known that the left and right hemispheres are anatomically and functionally asymmetrical (Bruder et al. 2017). Studies have shown that the bilateral ACC are also functionally distinct, with the left ACC being more involved in pain activation and response and the right ACC being more involved in pain-related emotion regulation (Etkin et al. 2011; Bliss et al. 2016; Xiao and Zhang 2018). Furthermore, increased FC between the left ACC and left insula of neuropathic pain patients is linked to pain sensitivity and pain memory processes (Sağ et al. 2019). Numerous studies have shown that the sACC is responsible for social processing and emotion regulation, and that the MTG is associated with memory and pain sensation (Drevets et al. 2008; Peyron et al. 2019; Petrides. 2023). Most recently, some studies have shown that the FC between the sACC.L and periaqueductal gray is positively correlated with pain intensity (Meeker et al. 2022). Consequently, the present findings emphasize the pivitol role of the sACC.L-related pain sensation subcircuit in the diverse symptoms of migraine.

Our study emphasizes the crucial role of the sACC.R in migraine pain emotion. One study exhibited that decreased FC between the right ACC and left medial prefrontal cortex (lmPFC) is strongly associated with emotional attenuation (Zhang et al. 2023). The most recent review emphasizes the involvement of the ACC in the emotional aspect of pain and its association with mood disorders, such as anxiety and depression (Journée et al. 2023). Furthermore, a previous study showed that the pACC/sACC.R regulates negative emotions such as fear and sadness (Peng et al. 2021), which may explain the abnormal FC of the pACC/sACC and the relationship between sACC.R and negative emotions in MWoA patients. In addition, our previous study on MM showed that the decreased FC between the sACC.R and DMN was closely related to SDS scores (Wang et al. 2022a). Thus, the above findings reveal an integral role of the sACC.R-pain emotion-related subcircuit in migraine.

In summary, we propose new insights into sACC.L-related pain sensations and sACC.R-related pain emotion subcircuits in patients with migraine. Based on the current findings, future intervention studies should focus on the key role of the sACC in migraine pain perception and pain emotion. Overall, the sACC may be a potential target brain region for the effects of interventions.

### Limitations

There were a few limitations in this study. First, there were more associations between brain regions and clinical indicators, making the correlation analysis relatively weak, and the results require further validation. Second, the specific physiological and pathological relationship between the left and right sACC and pain sensation and pain emotion in patients with migraine has not been fully clarified and warrants further exploration. And third, since differences in imaging protocols, software tools, and individual anatomical variability may significantly affect the accuracy and reproducibility of results, we should be cautious in interpreting the results.

## Conclusion

In conclusion, we combined structural and functional approaches to reveal the specific role of the ACC in pain sensation and emotions in patients with MWoA. Our findings provide novel insights into migraine abnormalities, shedding light on the distinct contributions of the sACC.L-related pain sensation subcircuit and sACC.R-related pain emotion subcircuit. These findings contribute to our understanding of the neuropathological significance and multidimensional treatment of migraine. Finally, they serve as a reference point for recognizing the link between structural and functional changes in the ACC subregions and symptoms, deepening our understanding of migraine and guiding clinical practice.

## Supplementary Material

SUPPLEMENTARY_bhae040
