# Supplementary material for: Structural and functional changes of anterior cingulate cortex subregions in migraine without aura: relationships with pain sensation and pain emotion
Source: Cereb Cortex. 2024 Feb 10;34(2):bhae040. doi: 10.1093/cercor/bhae040 (PMC10859245; doi:10.1093/cercor/bhae040)
Supplement: SUPPLEMENTARY_bhae040 [file supplementary_bhae040.docx]

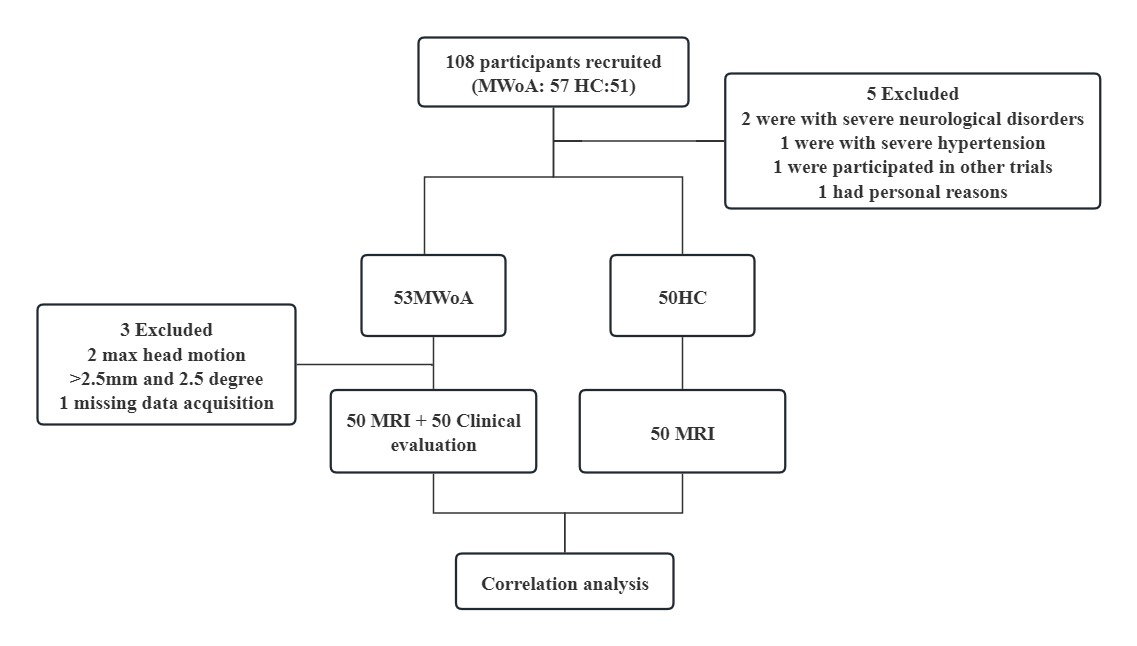


**Supplementary figure 1. Study Screening Flowchart.** Abbreviations: MWoA, migraine without aura; HC, healthy control; MRI, magnetic resonance imaging.
